# Immune Response of *Drosophila suzukii* Larvae to Infection with the Nematobacterial Complex *Steinernema carpocapsae–Xenorhabdus nematophila*

**DOI:** 10.3390/insects11040210

**Published:** 2020-03-28

**Authors:** Anna Garriga, Maristella Mastore, Ana Morton, Fernando Garcia del Pino, Maurizio Francesco Brivio

**Affiliations:** 1 Departament de Biologia Animal, Biologia Vegetal i Ecologia, Facultat de Biociències, Universitat Autònoma de Barcelona, Bellaterra, 08193 Barcelona, Spain; anna.garriga.oliveras@uab.cat (A.G.); ana.morton@uab.cat (A.M.); 2 Laboratory of Comparative Immunology and Parasitology, Department of Theoretical and Applied Sciences, University of Insubria, 21100 Varese, Italy; maristella.mastore@uninsubria.it

**Keywords:** *Drosophila suzukii*, immunity, entomopathogenic nematodes, *Steinernema carpocapsae*, *Xenorhabdus nematophila*, humoral defenses, cellular defenses

## Abstract

Entomopathogenic nematodes have been proposed as biological agents for the control of *Drosophila suzukii*, an invasive pest of small-stone and soft-skinned fruits. Larvae of the fly are susceptible to *Steinernema carpocapsae* infection but the reaction of immune defenses of the host are unknown. To determine the immune response, larvae were infected with *S. carpocapsae* and *Xenorhabdus nematophila* to evaluate the effector mechanisms of both humoral and cellular processes. The symbiont bacteria presented an inhibitory effect on the phenoloxidase cascade with a low level of melanization. Besides, *X. nematophila* activated the synthesis of putative antimicrobial peptides on the hemolymph of infected larvae. However, those peptides presented a lower antimicrobial activity compared to hemolymph from larvae infected with non-symbiont bacteria. *Xenorhabdus nematophila* avoided also the phagocytosis response of hemocytes. During in vitro and in vivo assays, *S. carpocapsae* was not encapsulated by cells, unless the cuticle was damaged with a lipase-treatment. Hemocyte counts confirmed differentiation of lamellocytes in the early phase of infection despite the unrecognition of the nematodes. Both *X. nematophila* and *S. carpocapsae* avoided the cellular defenses of *D. suzukii* larvae and depressed the humoral response. These results confirmed the potential of entomopathogenic nematodes to control *D. suzukii.*

## 1. Introduction

Entomopathogenic nematodes (EPNs) belonging to families Steinernematidae and Heterorhabditidae (Nematoda: Rhabditida) are obligate parasites of a wide range of insects [1]. These nematodes have a mutualistic relationship with a bacteria of the genera *Xenorhabdus* and *Photorhabdus* respectively, that helps to kill the insect [2]. The infective juveniles (IJs) enter the host through natural body openings or by penetrating the cuticle and release the bacteria [3]. The nematode-bacteria complex kills the host within 24 to 48 h through septicemia or toxemia [4]. Thus, nowadays, EPNs are used as biological control agents in the management of agricultural pests [5]. An important factor that affects the efficacy of EPNs is the immune response of the insect host [6]. The cuticle of the insects is the first defense against nematodes together with an intense grooming behavior [7]. When IJs penetrate through the cuticle into the hemocoel, physiological and immune defenses are activated in response to nematode presence [8,9]. Recognition of non-self, mainly based on the interaction between pathogen-associated molecular patterns and pattern-recognition receptors (PAMPs and PRRs), is crucial for the proper occurrence of cellular and humoral immune responses [10,11]. In insects, PAMPs and PRRs mediate the discriminatory step before triggering humoral responses, such as proPO system or antimicrobial peptide synthesis (AMPs). The proPO system is a complex enzymatic cascade responsible for the melanization reaction. This process leads to the production of melanin that can encapsulate invaders and opsonic factors enhancing immune reactions; moreover, drosophila phenoloxidases (PO) seem to play a role also in hemolymph clotting as a further defensive mechanism aimed to prevent the entry of nematodes and microorganisms [12,13,14]. Unlike the proPO system, which is rather well preserved and homogeneous among arthropod species, AMPs show different structural conformations among insects and various mechanisms to kill microorganisms [15].

PRRs also activate cellular responses like phagocytosis and encapsulation; phagocytosis is a conserved process mediated by hemocytes against various small targets including bacteria and yeast [16,17]. Instead, encapsulation is the main defense against the presence of multicellular targets, such as nematodes or endo-parasitoids. In the *Drosophila* family, three main types of hemocytes or immunocompetent cells (plasmatocytes, lamellocytes, and crystal cells) are found in the hemolymph and are responsible for the immune functions described [18]. Plasmatocytes represent the most abundant hemocytes and play a crucial role in target recognition, phagocytosis activity, and as promoters of encapsulation. These cells recall and differentiate to lamellocytes [19], which are involved in the formation of multi-layered capsules. The third cell population consists of crystal cells, which contain the enzymes of the proPO cascade and quickly degranulate in the presence of non-self [20].

Nevertheless, EPNs have developed strategies to evade and suppress the insect immune defenses during all stages of infection [6]. During a nematobacterial infection, three steps can be identified: in the early phase, IJs must evade and/or depress the host immune system just after entry. Afterward, in the midterm phase, symbiont bacteria are released and secret toxic compounds that contribute to killing the host. Finally, the long phase is the reproductive stage of nematodes [21]. Nemato-bacterial strategies are based on mimicry processes [22] or active suppression of host defenses [9]. *Steinernema carpocapsae* (Weiser) (Rhabditida: Steinernematidae) has been reported using mimic insect recognition proteins expressed in the epicuticle of IJs that evade detection [23,24]. This nematode can also damage immune defenses with proteolytic secretions, modulate proPO activity, and avoid encapsulation in different insect species [25,26,27]. In addition, its symbiont bacteria *Xenorhabdus nematophila* can cause general immunodeficiency using toxins that jointly with nematode defenses overcome the insect’s immune response [21]. Besides, Park and Kim [28] reported the ability of *X. nematophila* to avoid the activation of proPO cascade.

Our work is focused on *Drosophila suzukii* (Matsumura) (Diptera: Drosophilae) or spotted wing drosophila, the most important pest that attacks soft-skinned and small stone fruits causing significant losses to crops [29,30]. Despite chemical and culture methods are widely used, biological control of this fly has been attempted using natural enemies and entomopathogenic agents [31]. Studies with larvae of *D. suzukii* showed a strong immune response of encapsulation to parasitoid eggs of *Leptopilina heterotoma* Thompson (Hymenoptera: Figitidae) that discourages their use for controlling the pest [32,33]. Instead, pupal parasitoids, entomopathogenic fungi, and EPNs achieved better results controlling the fly under laboratory conditions [34,35,36,37]. Susceptibility of *D. suzukii* larvae was evaluated against different EPN species, as *S. carpocapsae, Steinernema feltiae* (Filipjev) (Rhabditida: Steinernematidae), and *Heterorhabditis bacteriophora* (Poinar) (Rhabditida: Heterorhabditidae) [36]. This study reported a high susceptibility of the fly after nematodes killed the larvae and reproduced inside them. Nevertheless, the immune response of *D. suzukii* to EPNs infection has not yet been studied.

Therefore, this work aimed to study the relationships between *D. suzukii* larvae and the nematobacterial complex *S. carpocapsae/X. nematophila*, from an immunological point of view. We evaluated humoral defenses, as the proPO system and lysozyme activity, the presence of antimicrobial peptides (AMPs) pool and its activity against bacteria. We analyzed the cellular response of *D. suzukii* larvae determining the phagocytosis and encapsulation ability of hemocytes and describing the immunoevasion strategies of *S. carpocapsae*.

## 2. Materials and Methods

### 2.1. Chemicals and Instruments

All reagents used in the assays were supplied by Sigma Chemicals (St. Louis, MO, USA), ICN (ICN Biomedicals, GmbH), Merck Millipore Ltd. (Tullagreen, Cork, Ireland), Bio-Rad Laboratories (Detroit, MI, USA). The equipment was supplied by Bio-Rad Laboratories (Detroit, MI, USA) and Celbio Spa (Milan, Italy, EU). Centrifugations were carried out with a SIGMA 1-14 (SciQuip Ltd., Newtown, Wem, Shropshire, UK) and an Eppendorf 5804R (Eppendorf, AG, Hamburg, Germany). Spectrophotometric assays were performed with a Jasco V-560 spectrophotometer (Easton, MD, USA). All materials, buffers, and solutions were autoclaved or filtered with 0.22 µm Minisart filters (Sartorius, Goettingen, Germany). For microscopy observations, a microscope Olympus IX-51 epifluorescence connected to a Nikon digital camera was used.

### 2.2. Insects and Nematodes

The third stage of *D. suzukii* larvae used for all assays was obtained from a laboratory culture of specimens collected in Catalonia (NE Spain) in 2012. These insects were reared on a *Drosophila* diet [37] and maintained in a climate chamber at 25 °C, 45% RH, and a 12:12 h photoperiod.

The EPN species used in this study was *S. carpocapsae* (B14) isolated from urban garden soil in Barcelona (Catalonia, NE Spain). Nematodes were reared at 25 °C in the last instar larvae of *Galleria mellonella* (Lepidoptera: Pyralidae) according to the method described by Woodring and Kaya [38]. The IJs emerging from insect cadavers were collected with modified White traps [39] and stored in sterile tap water in culture flasks at 9 °C for a maximum of two weeks. Before use, IJs were acclimatized at room temperature for 3 h and their viability was checked by observation of movement under a stereomicroscope. IJs were selected, washed several times with sterile phosphate buffer (PBS) (8.0 g NaCl, 0.2 g KCl, 1.44 g Na_2_HPO_4_, 0.24 g KH_2_PO_4_ per liter, pH 7.4), and centrifuged at 100× g for 2 min at 20 °C. Assays were performed using alive, dead and lipase-treated dead nematodes, to evaluate the role of the body surface of *S. carpocapsae* in the immune-evasive processes.

To kill the nematodes, they were frozen at −20 °C for at least 5–6 h in PBS plus 20% of glycerol. To modify the cuticular lipid layer, killed nematodes were treated with 50 µl of lipase (10 U/µL in 30 mM of Tris-HCl, pH 8), at 37 °C, for 90 min; after the enzymatic digestion, nematodes were washed several times in sterile buffer.

### 2.3. Bacteria Cultures and Infection Protocol

To culture the symbiotic bacteria, *X. nematophila* were isolated from *S. carpocapsae* according to the method of Park and Kim [40]. *X. nematophila* Green Fluorescent Protein (GFP-labeled) was also kindly provided by the laboratory of Prof. Givaudan (University of Montpellier, France). Bacteria were inoculated into liquid broth (30g/L Tryptic Soy Broth) and incubated at 30 °C in agitation, overnight under dark conditions. The culture was grown to an optical density (OD) of 0.6; the growth curve was measured spectrophotometrically at 600 nm. Bacteria were centrifuged at 1700× g for 20 min and bacterial pellets were washed several times with PBS. Aliquots at different concentrations were prepared for corresponding assays.

Cultures of *Escherichia coli* (C1a), *Bacillus subtilis* (ATCC N° 6633), and *Micrococcus luteus* (ATCC N° 4698) were prepared for positive stimulation of *D. suzukii* larvae. Bacteria were inoculated and grown in Luria broth (20 gr/L) at 37 °C and 30 °C for *M. luteus* in agitation, overnight under dark conditions. Bacteria were grown up to 0.6 OD (10^9^ CFU/mL) and were centrifuged at 1500× g for 15 min at 20 °C. After centrifugation, the pellets were washed several times in PBS. Final bacterial concentrations and species mixtures were reported in each assay.

### 2.4. Hemolymph Collection

To extract the hemolymph, third stage *D. suzukii* larvae were washed in PBS and 70% ethanol solution and anesthetized at 4 °C. Depending on the assay, 20 to 40 larvae were cut in the dorsal region with a microsurgical scissor and transferred in PCR tubes properly prepared for the procedure. The bottom of the tube was holed several times with a needle and inserted into a 0.5 mL Eppendorf so that during centrifugation, the hemolymph was collected in the large tube. Samples were centrifuged at 250× g for 5 min at 4 °C to collect whole hemolymph containing hemocytes. For humoral assays (Section 2.6, Section 2.7), the supernatant, corresponding to a cell-free fraction (CFF), was recovered, centrifuged at 720× g and a few phenylthiourea (PTU) crystals were added; all the humoral immunity assays were carried out according to the methods described in Mastore and Brivio [41]. For cellular assays (Section 2.8, Section 2.9), 10 µL of PTU (from a saturated stock solution) were added to the whole hemolymph to prevent unwanted activation of phenoloxidase.

### 2.5. proPO System Relative Activity in the Host Hemolymph

To test the proPO activity of *D. suzukii* larvae against bacterial infection, phenoloxidase relative activity was analyzed in the hemolymph by spectrophotometric analysis with L-Dopa as a substrate.

Larvae of *D. suzukii* were washed with PBS and anesthetized by exposure to cold; then, they were infected with bacteria using a pricking method. Pricking consisted of a puncture with a wolfram needle soaked in bacteria pellet obtained from a suspension of 10^9^ CFU/mL. Four different treatments were evaluated: naive larvae, control pricked larvae, *X. nematophila* infected larvae, and *E. coli*/*B. subtilis* infected larvae. A mixture of *E. coli* and *B. subtilis* 1:1 (v/v) was prepared. Larvae were kept at rearing conditions until hemolymph was collected 30 min after any treatment. After the extraction, the total protein content was determined and the reaction volumes were normalized according to concentration, 2.5 µL of hemolymph was added in 1 mL of L-Dopa buffer (8 mM L-Dopa in 10 mM Tris-HCl, pH 7.2). The increase of absorbance was recorded at 490 nm (Δ*A*_490_ 5 min^−1^) at 25 °C, by a double-beam Jasco V-560 spectrophotometer (Easton, MD, USA) using L-Dopa buffer as blank. For each treatment, 20 larvae were used, and the experiment was repeated 5 times.

### 2.6. Lysozyme Activity after Bacterial Infection

To evaluate the activity of lysozyme in naïve and infected larvae of *D. suzukii*, the turbidimetric method was used with *Micrococcus lysodeikticus* as a substrate. This method is based on the decrease of absorbance due to the lysozyme-induced cell lysis determined as a unit of lysozyme produced in one minute a variation of OD 450 nm of 0.001. Larvae of *D. suzukii* were infected with *X. nematophila* and *E. coli*/*M. luteus* mixture 1:1 (v/v) at a concentration of 10^9^ CFU/mL, using the pricking method. After infection, larvae were kept with diet at 25 °C for 24 h. Then, hemolymph samples were extracted from larvae, cells were removed and CFF samples, added with PTU crystals, were diluted with PBS (1:10). For the analysis, 0.45mg/mL of *M. lysodeikticus* lyophilizate were resuspended in 0.3 M PBS, pH 6.8 and mixed for 1 min at room temperature. For each treatment, 60 µL of diluted CFF were added to 90 µL of *M. Iysodeikticus* suspension into a well of 96-MicroWell™ plate. As a control, a suspension of *M. lysodeikticus* (90 µL) plus PBS (60 µL) was used, and a PBS (90 µL) plus hemolymph sample (60 µL) was used as a blank for each treatment. The activity of lysozyme was assessed immediately using a microplate-Reader (Bio-Rad, Hercules, CA, USA) and absorbance variations were recorded every min for 10 min at 450 nm. For each treatment, 40 larvae were used, and the experiment was repeated three times.

### 2.7. Analysis by Tricine-PAGE and Activity of AMPs

We analyzed changes in proteins and peptides patterns in hemolymph samples (especially in CFF) of *D. suzukii* larvae using the electrophoretic separation by Tricine-SDS-PAGE methodology [42]. Moreover, the antimicrobial activity of these CFF samples was evaluated with bacteria growth tests.

Larvae of *D. suzukii* were infected using the pricking method with a bacterial suspension of *X. nematophila* and *E. coli*/*M. luteus* mixture 1:1 (v/v) at a concentration of 10^9^ CFU/mL. Larvae were kept in rearing conditions for 24 h. Then, the hemolymph from naïve infected *X. nematophila* and *E. coli/M. luteus* infected larvae was extracted. From these CFF samples, we carried out the analysis for the presence of putative AMPs molecules and the evaluation of the antimicrobial activity.

For the electrophoretic separations, hemolymph was fractioned by Amicon^®^ Ultrafilters (Millipore, Burlington, MA, USA) cut-off 30 KDa and precipitated with trichloroacetic acid (20% V/V). Then, samples were resuspended in 1× Tricine-PAGE sample buffer [42] and denatured for 5 min at 100 °C. Electrophoresis was carried by a vertical PROTEAN^®^ II xi Cell (Bio-Rad) at 50 V (constant voltage) overnight. Protein patterns were detected by Silver Staining.

For the antimicrobial activity, CFF samples were centrifuged at 1700× g for 15 min and fractioned <30 kDa; *E. coli*, *M. luteus*, and *X. nematophila* cultures were diluted to a final concentration of 10^6^ CFU/mL with culture broth. For each treatment, 20 μL of CFF sample were added to 180 μL of bacteria culture. To evaluate the expected bacterial growth, 20 μL of PBS were added to the bacteria culture (180 μL). All samples were incubated for 3 h under shaking at the optimal growth temperature of the tested bacterium. After incubation, 100 µL of each sample was placed in a well of a 96-MicroWell™ plate and samples were serially diluted with phosphate buffer (61.4 mM K_2_HPO_4_, 38.4 mM H_2_PO_4_). Each dilution was plated on solid agar and incubated for 24 h more. Finally, bacteria colonies were counted. The antibacterial activity in hemolymph samples was intended as a percentage of bacterial survival compared with the control (bacterial suspension incubated without *D. suzukii* hemolymph). The final concentration of hemolymph total proteins used in the antimicrobial activity tests was 3.3 µg/µL. For each analysis and treatment, hemolymph of 40 larvae was extracted and the experiment was done three times.

### 2.8. Phagocytic Activity Assay

Phagocytic activity of *D. suzukii* hemocytes was evaluated both in vivo and in vitro in the presence of *X. nematophila*. For the in vivo assay, 60 larvae of *D. suzukii* were infected with *X. nematophila*-GFP (10^4^ CFU/50nL). Microinjections were performed by a Drummond Nanoject II nanoliter injector (Drummond Scientific Company, PA, USA). As a positive control, 60 larvae were injected with 50 nl (1 mg/mL) of a suspension of pHrodo^®^ Red *Staphylococcus aureus* Bioparticles^®^-Conjugate (Life Technologies, Carlsbad, CA, USA). Larvae were kept in rearing conditions and hemolymph was collected after 2 h. Extracted hemolymph with hemocytes was added to Schnëider medium in 96-MicroWell™ (Thermofisher Scientific, Waltham, MA, USA) plates (final concentration 2 × 10^5^ cells/mL) and incubated for 30 min at 25 °C in the dark. To evaluate the phagocytic activity of hemocytes, after adhesion, cells were observed under a fluorescence microscope.

For in vitro assay, hemolymph of 40 naïve larvae was extracted and hemocytes were plated in 96-MicroWell™ plates at a concentration of 2 × 10^5^ cells/mL in Schnëider medium. To allow cells to adhere to the substrate, plates were incubated for 1 h at 25 °C in the dark. Then, 5 µL (10^3^ CFU) of *X. nematophila*-GFP or *S. aureus*-pHrodo^®^ were added to cells and incubated for 4 h at 25 °C. After incubation, cells were observed under a fluorescence microscope to assess the phagocytic activity of hemocytes. Both experiments were performed three times.

### 2.9. In Vitro Encapsulation Assay

We assessed the ability of the hemocytes of *D. suzukii* larvae to recognize and encapsulate *S. carpocapsae* with an in vitro assay performed with three treatments. Alive IJs were used to test the physiological response of the host immune cells to the presence of the nematode. To exclude that unrecognition could be caused by active secretions, cold-killed nematodes were incubated with hemocytes. Besides, to investigate a possible mimetic function of the body-surface of *S. carpocapsae*, we modified the nematode cuticle using lipase enzyme treatment.

Hemocytes from naïve larvae were extracted and plated with Schnëider medium in a 96-MicroWell™ plate (2 × 10^5^ cells/mL). Then, 5–10 nematodes per treatment (alive, cold-killed, or lipase-treated dead) were added to the microwells and incubated in a climate chamber at 25 °C. As positive control to evaluate encapsulation capability, we added 10–15 agarose beads (DEAE Sepharose^®^) into wells. Encapsulation processes were monitored at different times along 24 h with an inverted fluorescence microscope. Each treatment was performed three times.

### 2.10. Hemocytes Populations Count after Natural Infection

To evaluate the in vivo ability of *D. suzukii* hemocytes to recognize and isolate *S. carpocapsae*, we carried out encapsulation assays by a natural infection. Concurrently, total and differential cell counts (plasmatocytes, lamellocytes, and crystal cells) were performed to assess any possible variation of cell populations after nematode infection.

Ten larvae were placed in a Petri dish (3 cm diameter) filled with filter paper and exposed to *S. carpocapsae* (50 IJs cm^−2^) for 20 h. The control treatment was carried out with sterile tap water. To detach any adherent cells from the hemocoel cavity, larvae were gently brushed, and then washed with PBS [43]. An incision was done behind the mandibles and all hemolymph (approximately 2 µL) was bled in 48 µL of buffered PTU (saturated PTU diluted 1:4 in PBS). After bleeding, hemolymph content of each separated larvae was observed under the microscope, to detect the presence and number of nematodes inside the larvae, cell encapsulation processes, and the possible presence of symbiotic bacteria released in the hemolymph. Depending on the phase of infection, larvae were divided into two groups: early infection (only nematodes were present) or midterm infection (bacteria were released). Total hemocyte count was made applying the diluted hemolymph into the hemocytometer (Neubauer chamber, Brand^®^). The cell counts were performed immediately by determining the total number of cell populations and the number of different types of hemocytes identified. For each treatment, ten larvae were evaluated, and the experiment was done twice.

### 2.11. Statistical Analysis

To analyze the differences in enzymatic activity of proPO and lysozyme, a General Linear Model (GLM) analysis has been used to ascertain differences among the tested samples. GLM was also used to elucidate differences among CFF samples of their antimicrobial activity against different bacterial cultures. Hemocyte counts were measured as the number of cells/µL of hemolymph and differences among treatments were analyzed with GLMs. For all experiments, when the GLM was significant, differences were evaluated by Tukey test and means without transformation (±SD) are presented. All statistical analyses were performed with the R studio software (version 1.0.153) [44], and any comparison was considered significant if the *p*-value was < 0.05.

## 3. Results

### 3.1. proPO System Relative Activity in the Host Hemolymph

**Figure 1:**
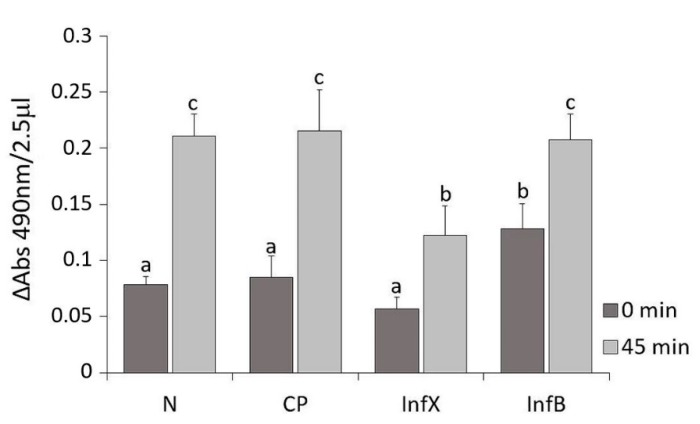
Phenoloxidase relative activity in the hemolymph of *D. suzukii* shown as the mean ± SD increase of absorbance, recorded at 0 and 45 min (= 490 nm), in hemolymph samples from naïve (N), control pricked (CP), *X. nematophila*-infected (InfX) and *E. coli/B. subtilis*-infected (InfB) larvae. Different letters indicate statistical significance differences between hemolymph samples (*p* < 0.05).

The relative activity of phenoloxidase enzyme in *D. suzukii* samples was evaluated by recording spectrophotometrically the formation of dopachrome (Figure 1, Figure S1, and Table S1).

Samples from naïve larvae (N), control pricked larvae (CP), infected larvae with *X. nematophila* (InfX) and infected larvae with *E. coli*/*B. subtilis* (InfB) were analyzed. The absorbance values recorded showed no significant difference between naïve and control pricked larvae. These values represent the physiological activity of the enzyme until the formation of all dopachrome (time 45 min). Larvae infected with *X. nematophila* presented similar values to both naïve and control larvae at 0 min, but the presence of symbiotic bacteria produced an inhibition of phenoloxidase activity 45 min after the start of reaction. The enzyme showed a significantly minor activity compared to the other hemolymph samples (GLM: *F* = 42, *df* = 7, *p* < 0.001). In contrast, the proPO system of *D. suzukii* larvae resulted in *E. coli* and *B. subtilis* infection showing a significantly high increase of absorbance at 0 min after hemolymph extraction.

### 3.2. Lysozyme Activity after Bacterial Infection

The assays of lysozyme activity in the hemolymph of naïve *D. suzukii* larvae showed extremely low activity (1 × 10^−1^ Units mL^−1^). Moreover, those infected with *X. nematophila* also presented a low level of activity (1.1 × 10^−1^ Units mL^−1^). Only after infection with *E. coli* and *M. luteus*, a slight increase in the enzyme activity was recorded (1.3 × 10^−2^ Units mL^−1^). Nevertheless, lysozyme activity in the hemolymph of *D. suzukii* larvae was not significantly stimulated by the infection of *X. nematophila* or the bacterial mixture of *E. coli* and *M. luteus* (GLM: *F* = 0.12, *df* = 2, *p* = 0.81).

### 3.3. Analysis by Tricine-PAGE and Activity of AMPs

**Figure 2:**
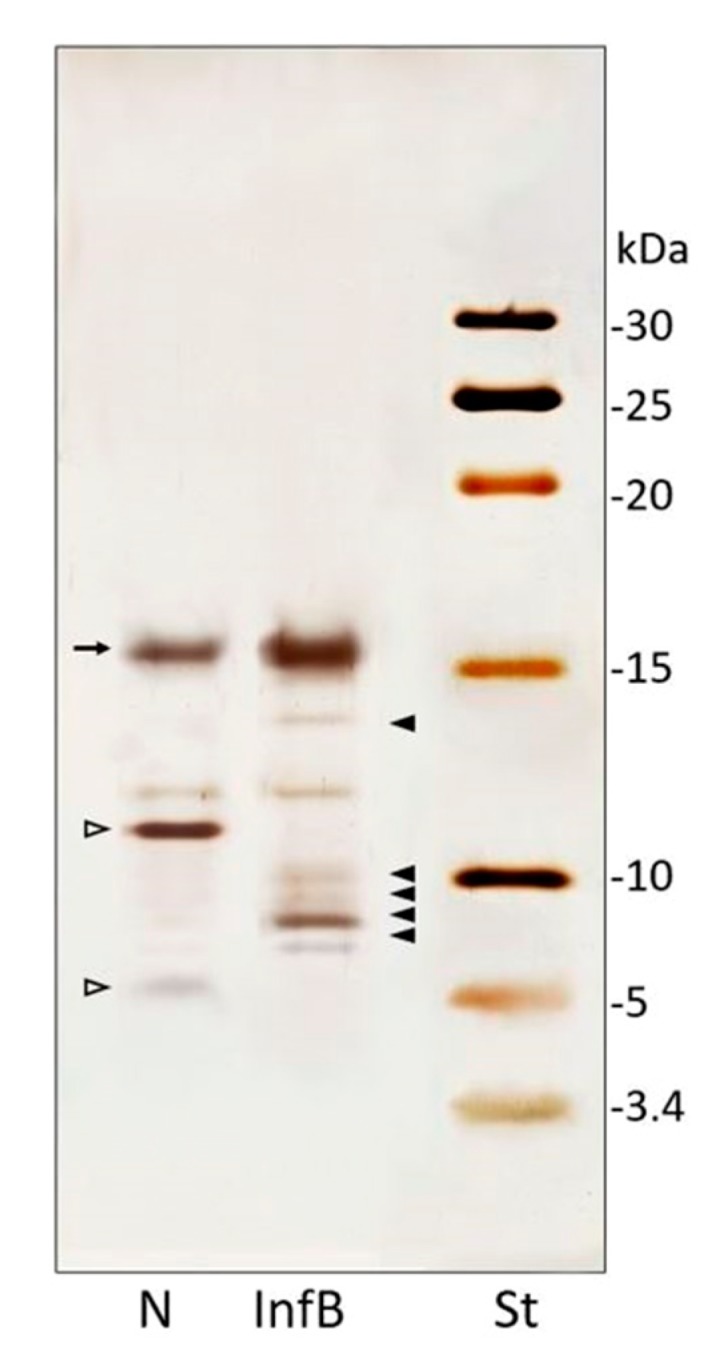
Tricine-SDS-PAGE (16%) of hemolymph samples. Patterns of low molecular weight proteins (<30 kDa) of hemolymph from naïve (N) and *E. coli/M. luteus*-infected (InfB) larvae; standard molecular weights marker (St). Full arrowheads indicate newly synthesized bands and the arrow an increased band, observed after bacterial infection. Empty arrowheads indicated disappeared peptides after bacterial infection.

Electrophoretic separation of fractioned CFF (<30 kDa) showed proteins and peptide patterns of hemolymph samples from naïve and infected larvae (Figure 2).

In naïve larvae CFF, four main bands were present and two of them disappear in the infected larvae sample (Figure 2, empty arrowhead). Moreover, peptides pattern from *D. suzukii* larvae infected with *X. nematophila* (Figure S2) and infected with the mixture of *E. coli*/*M. luteus* showed newly synthesized or quantitatively increased peptides of low molecular weight (Figure 2, full arrowheads). Five main bands, ranging from 5 to 16 kDa, absent in CFF from naïve larvae (Figure 2, N), were observable in samples from treated larvae (Figure 2, InfB). Moreover, a band of about 16 kDa, increased in samples from infected larvae (Figure 2, InfB, arrow). The infection with *X. nematophila* (Figure S2) and *E. coli*/*M. luteus* resulted in the disappearance of two peptides (Figure 2, N, empty arrowheads).

**Figure 3:**
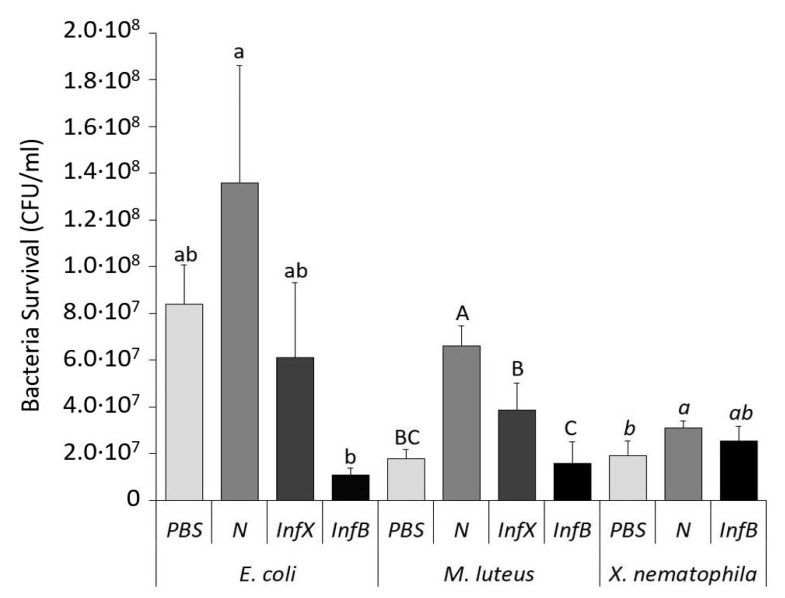
Antimicrobial activity of *D. suzukii* hemolymph after bacterial infection evaluated through co-incubation with *E. coli*, *M. luteus*, and *X. nematophila*. Host hemolymph samples were from PBS-control (PBS), naïve larvae (N), infected with *X. nematophila* (InfX), and with *E. coli/M. luteus* (InfB). Survival of *E. coli, M. luteus*, and *X. nematophila* are shown as the mean ± SD of CFU/ml. Different letters on the bars represent statistically significant differences among each antimicrobial sample. GLM of *E. coli: F* = 8.39, *df* = 3, *p* = 0.007; GLM of *M. luteus: F* = 21.73, *df* = 3, *p* < 0.001; GLM of *X. nematophila*: *F* = 3.66, *df* = 2, *p* = 0.091.

After identification of the peptides pattern, the antimicrobial activity in the hemolymph was analyzed by co-incubation of CFF samples with bacterial cultures of *E. coli*, *M. luteus*, and *X. nematophila*. Bacterial growth (CFU/mL) represented a negative correlation of AMPs activity present in larvae hemolymph of naïve, infected with *X. nematophila* and infected with *E. coli*/*M. luteus* (Figure 3, Table S2).

As there was significant higher proliferation of all bacteria incubated with naive hemolymph compared to buffer incubation (PBS), we evaluated the bacterial mortality percentage assuming as 100% the data obtained with naive hemolymph samples (Table S3). CFF samples from larvae infected with *X. nematophila* reduced significantly the bacterial growth of *E. coli* to 6.11 × 10^7^ CFU/mL and *M. luteus* to 3.86 × 10^7^ CFU/mL. These results showed a degree of antimicrobial activity in hemolymph against both bacteria strains with 55.0% and 41.5% of mortality respectively (Table S3). However, CFF samples of *E. coli/M. luteus*-infected larvae presented a drastic antimicrobial effect against *E. coli* (1.06 × 10^7^ CFU/mL) causing mortality of 92.2% and 76.1% against *M. luteus* (1.58 × 10^7^ CFU/mL). In contrast, when tested on *X. nematophila* the recorded mortality was markedly lower (17.2%) (Table S3). Thus, CFF from larvae infected with *X. nematophila* showed antimicrobial activity against the three bacterial cultures tested, although lower than the obtained after *E. coli/M. luteus* infection.

### 3.4. Phagocytic Activity Assay

During in vivo phagocytosis assay, hemolymph of *D. suzukii* larvae injected with *X. nematophila*-GFP was extracted and monitored by fluorescence microscopy.

**Figure 4:**
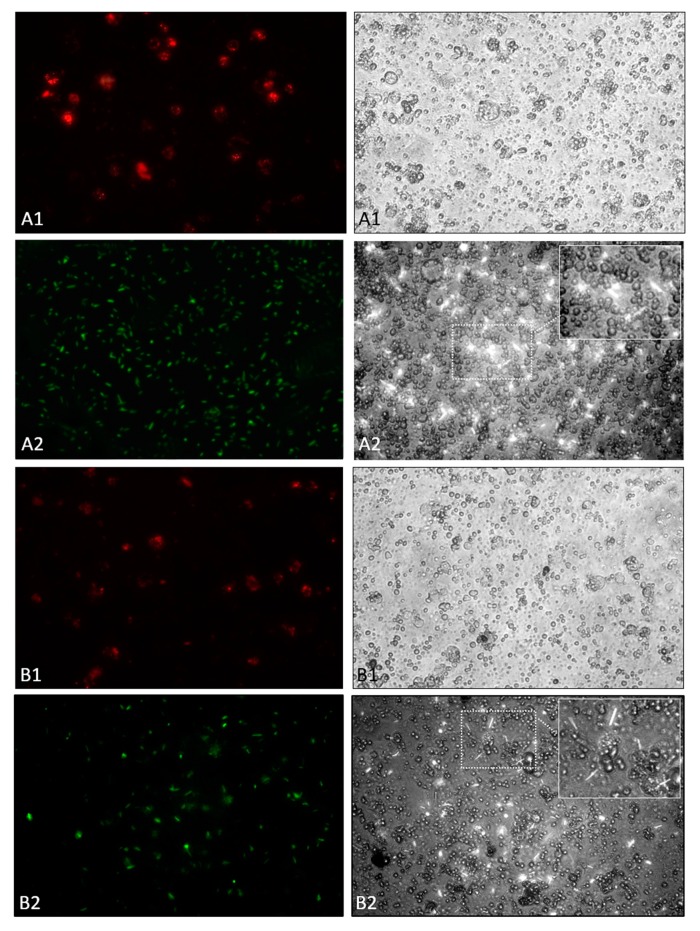
In vivo (**A**) and in vitro (**B**) phagocytic activity of *D. suzukii* larvae hemocytes against bacteria. (**A1**) in vivo assay at 2 h with *S. aureus*-pHrodo^TM^ BioParticles^®^; (**A2**) in vivo assay at 2 h with *X. nematophila*-GFP, inside A2 right an enlargement of the central area of the image. (**B1**) in vitro assay at 4 h with *S. aureus*-pHrodo^TM^ BioParticles^®^; (**B2**) in vitro assay at 4 h with *X. nematophila*-GFP, inside B2 right an enlargement of the central area of the image. All images on the right are bright field of the respective fluorescence micrographs on the left. Images magnification is 400×.

To evaluate the phagocytosis capability of hemocytes, we carried out in vivo assays using *S. aureus* pHrodo^®^-conjugated which fluorescence was activated only at acidic pH (inside phagolysosomes). *Staphylococcus aureus*-pHrodo was effectively engulfed by host cells as confirmed by the intense fluorescence of the probe (pHrodo) (Figure 4, A1, left). Besides, in the bright field, hemocytes of *D. suzukii* were properly adhered to the substrate and are viable and showing a morphology typical of cells that have engulfed (Figure 4, A1, right). Otherwise, hemocytes seem to be unable to phagocytize *X. nematophila*-GFP (Figure 4, A2, left); both the elongated rod shape and swipes of the symbiont bacteria, indicate their extracellular localization. The entomopathogenic bacteria, in addition to not being phagocytized, seem to have cytotoxic effects on the host hemocytes, affecting the morphology of the cells (Figure S3). Besides, Figure 4 (A2, right) shows the micrograph obtained by combining fluorescence and phase contrast, in which the extracellular location of the symbiont bacteria can be detected.

Hemocytes of *D. suzukii* larvae established in the in vitro cultures showed comparable results to those obtained in vivo; *S. aureus*-pHrodo was efficiently phagocytized (Figure 4, B1, left) and hemocytes show the morphology of cells that are engulfing (Figure 4, B1, right). When *X. nematophila*-GFP was added to the culture, hemocytes were not able to phagocyte the symbiont bacteria as confirmed by the observation of Figure 4, B2, left and right.

### 3.5. In Vitro Encapsulation of S. carpocapsae

**Figure 5:**
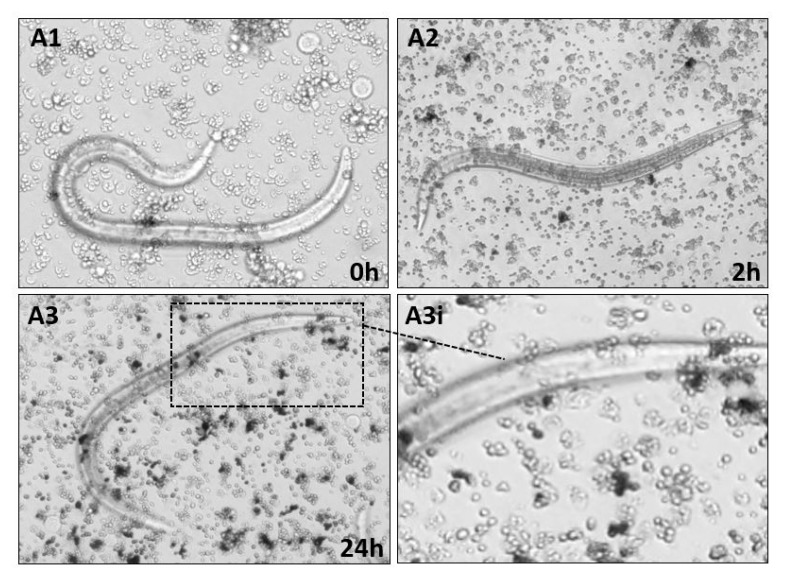

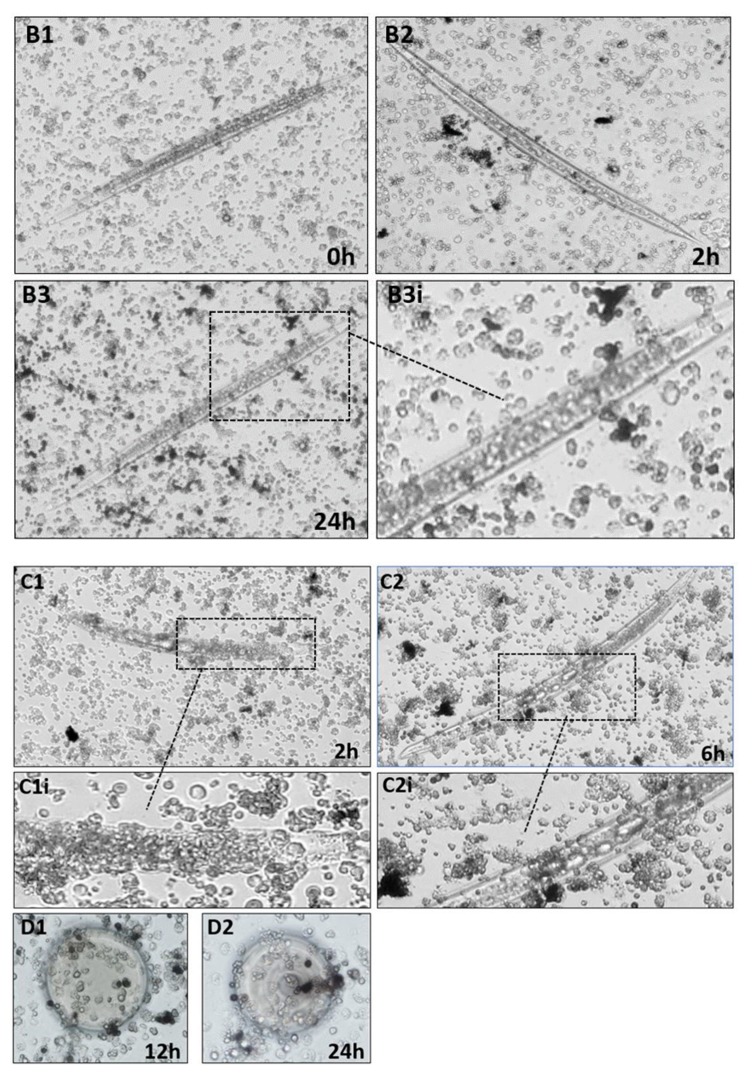
(**A**,**B**) In vitro encapsulation assays of S. carpocapsae by hemocytes of D. suzukii larvae. Nematodes were co-incubated with hemocytes. From top to bottom: alive nematodes at 0 h (**A1**), 2 h (**A2**) and 24 h (**A3**), A3i inset is an enlargement of A3. Cold-killed nematodes at 0 h (**B1**), 2 h (**B2**), and 24 h (**B3**), B3i inset is an enlargement of B3. A1 (200×); A2, A3, B1, B2, B3 (100×). (**C**,**D**). In vitro encapsulation assay of lipase treated dead nematodes at 2 h (**C1**) and 6 h (**C2**), C1i and C2i are enlargements of the respective images. In vitro encapsulation of agarose beads, at 12 h (**D1**) and 24 h (**D2**). C1 and C2 (100×); D1 and D2 (200×).

Cellular encapsulation of nematocomplexes was assessed using in vitro long-term co-incubation. Hemocyte response against *S. carpocapsae* alive, dead (cold-killed), or surface lipase-treated was monitored under an inverted microscope for 24 h. We observed that alive nematodes were not recognized nor encapsulated by hemocytes even after 24 h (Figure 5A,B: A1,A2,A3,A3i).

Moreover, no cellular processes directed against *S. carpocapsae* were evident. We performed the same experiment with cold-killed nematodes to exclude the influence of active secretions. As seen before, hemocytes were unable to recognize dead IJs of *S. carpocapsae* and no encapsulation processes were observed also at long times (Figure 5A,B: B1,B2,B3,B3i). When lipase-treated nematodes were used, it was possible to observe a reactivity of hemocytes adhering to the body surface of the treated nematodes (Figure 5C,D: C1 and C1i, C2 and C2i). After 2 h (Figure 5, C1), numerous layers of cells were attached to the cuticle and would contribute to the building of the cellular capsule. The abiotic targets (Sepharose DEAE microbeads) used as a control presented cellular reactivity with hemocytes adhered to beads and the presence of melanin clots at 12 and 24 h after incubation (Figure 5C,D: D1 and D2, respectively).

### 3.6. Hemocytes Populations Count after Natural Infection

Natural infection of *S. carpocapsae* in *D. suzukii* larvae showed no cellular encapsulation processes of nematodes, as we observed in the previous in vitro assay. IJs were found free without attached cells on the cuticle, even if *X. nematophila* was released in the hemolymph. Hemocyte counts were performed when the mean number of nematodes inside larvae was 5.9 ± 4.3 IJs. Counts were avoided when the nematode number was over 15 IJs because the massive entry of nematodes into small larvae like *D. suzukii* generate tissue damage quickly. Infected larvae were separated in the early phase or midterm phase of infection if bacteria were released. Larvae in the early phase were mostly still alive (95%), while 30% of larvae were alive in the midterm phase.

Total hemocytes count showed a high number of immunocompetent cells (2.63 × 10^4^ cells/µl) in naive *D. suzukii* larvae hemolymph (Table 1).

**Figure 6:**
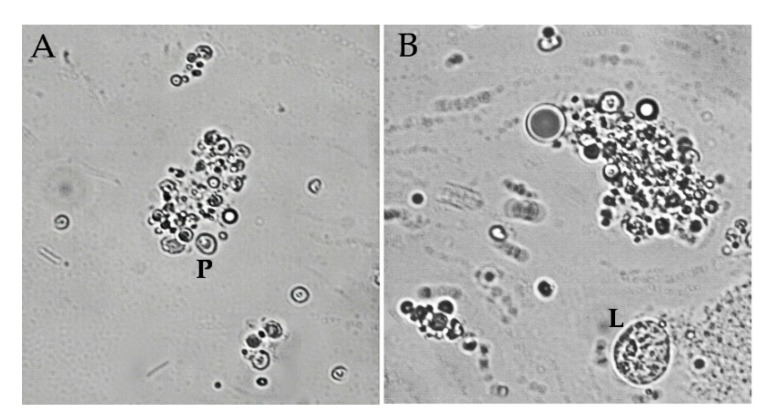
(**A**) Plasmatocytes (P) aggregation in in vivo encapsulation assay during the midterm phase of infection. (**B**) Aggregation of hemocytes next to a circulant lamellocyte (L). Image magnification is 400×.

Nevertheless, infected larvae showed a significant decrease in total hemocytes in the early and midterm phase of infection (GLM: *F* = 14.26, *df* = 2, *p* = 0.000). Plasmatocytes constituted the major fraction of hemocytes and presented a decrease in both phases of infection, as total hemocytes (GLM: *F* = 16, *df* = 2, *p* = 0.000). In naïve larvae, lamellocytes represent a very small fraction of immune cells but during the early phase of infection, the amount of lamellocytes increased significantly (GLM: *F* = 93.99, *df* = 2, *p* = 0.000). During the midterm phase, the bacteria were released, and we observed the attachment of lamellocytes and plasmatocytes with themselves, becoming big aggregations (Figure 6A,B). Due to the difficulty of counting only free cells were considered. In consequence, lamellocytes level decreased showing no significant difference with naïve value.

Nevertheless, IJs were not found close to these cell aggregations. The only hemocytes population that seemed unaffected by nematode infection were the crystal cells. Neither nematode nor bacterial infection altered the number of crystal cells that remained constant in all assays (GLM: *F* = 1.71, *df* = 2, *p* = 0.188).

## 4. Discussion

*D. suzukii* represents a major threat to berry, cherry, and strawberry production as a globally invasive pest. This fly belongs to the *melanogaster* subgroup, as well as *Drosophila melanogaster* (Meigen) (Diptera: Drosophilidae), which immune response to EPNs is widely studied as an insect model [18,43]. In contrast, few immunological studies have been conducted with *D. suzukii* and only focused on the parasitoid response. Poyet et al. [33] described a strong immune reaction against the parasitoid *L. heterotoma*, which leads to the encapsulation of wasp eggs. Even if *D. suzukii* larvae and adults are susceptible to nematodes [36], no immunological studies on the relationships between these pests and EPNs have been made before; consequently, we evaluated humoral and cellular responses upon infection of *S. carpocapsae* and *X. nematophila*.

The fastest defense of insect larvae is the activation of the phenoloxidase cascade. The symbiont bacteria *X. nematophila* showed an inhibitory effect on the host proPO system, with levels of phenoloxidase activity lower than naïve larvae. In contrast, *E. coli* and *B. subtilis* infection registered higher activity. These results agree with those obtained comparing the melanization rate of *D. melanogaster* infected with symbiont and axenic *S. carpocapsae* [45]. These authors reported that in the presence of *X. nematophila*, levels of melanization were significantly lower than using the axenic nematode. The inhibitory role of this symbiont bacteria and involvement of the eicosanoid pathway has been also reported in some lepidopteran species such as *Spodoptera exigua* (Hübner) (Lepidoptera: Noctuidae) and *Plutella xylostella* L. (Lepidoptera: Plutellidae) [28,46]. These authors related the secretion of a phospholipase A_2_ (PLA_2_) inhibitor with the suppression of the proPO activation in hemolymph and the alteration of cellular response. Despite no homologous genes were identified in *D. melanogaster*, Scarpati et al. [47] found other genes involved in eicosanoid pathway that could be functional equivalents.

The assay of lysozyme activity in naïve larvae of *D. suzukii* showed an extremely low activity compared to other insect species such as *Galleria mellonella* (Lepidoptera) 2.28 × 10^3^ Units mL^−1^ or *Sarcophaga africa* (Diptera) 1.04 × 10^2^ Units mL^−1^ [48]. There was no significant difference when larvae were stimulated by bacteria. Thus, lysozyme activity was not triggered by the infection *X. nematophila* nor *E. coli/M. luteus*. In agreement with our results, the *Drosophila* genus showed to have mainly digestive lysozyme and an insignificant amount of enzyme in the hemolymph for immune defense [49].

Along with phenoloxidase and lysozyme enzymes, AMPs perform a key role in humoral defense against bacterial infection. When AMPs presence was assessed by electrophoretic separations, both bacterial infections showed comparable band patterns with newly synthesized bands compared to naïve larvae. However, the antimicrobial activity test revealed a higher activity of the bacterial mixture infected larvae as opposed to larvae infected with *X. nematophila*. These results suggested that there was a synthesis of putative AMPs after symbiont infection. Nevertheless, larvae infected with *X. nematophila* showed lower activity with respect to that from larvae infected with non-entomopathogenic bacteria. With the conducted assay, we are unable to determine the causes, although these results could suggest an active mechanism of *X. nematophila* to disable the activity of those peptides. Some authors attributed to symbionts bacteria the ability to down-regulate AMPs genes in *S. exigua* or *Rynchophorus ferrugineus* Olivier (Coleoptera: Curculionidae) [50,51,52]. However, Peña et al. [45] reported in *D. melanogaster* an increased gene expression of AMPs in response to *X. nematophila* infection, over an infection of *S. carpocapsae*. Indeed, genes could be up-regulated in *D. suzukii*, as we detected the presence in the hemolymph of some peptides in the molecular mass range of AMPs. Although, the symbiont bacteria are known to release cytotoxic proteins and antimicrobial inhibitors to block the function of the peptides [53].

In addition to the analysis of humoral responses, we have also observed interference of the nematobacterial complex with the host cellular responses. As observed, either in in vivo or in vitro assays, *X. nematophila* was able to avoid phagocytosis response by the host immunocompetent cells; the phagocytic capability of the hemocytes was ascertained by the assays with *S. aureus*. Shrestha and Kim [54] reported in *S. exigua* larvae that the disruption of phagocytosis and avoidance of cell reaction to bacteria was caused by the synthesis of PLA_2_ inhibitors by *X. nematophila*. Similar results were also obtained with *Manduca sexta* L. (Lepidoptera: Sphingidae) larvae, where *E. coli* was engulfed more than *Photorhabdus luminescens* (the symbiont bacteria of *H. bacteriophora*) [55]. Both symbionts bacteria use inhibitor of the PLA_2_; likewise, in *D. suzukii* larvae, *X. nematophila* could implement the same strategy to avoid the host phagocytosis. Our data showed the ability of *S. carpocapsae* to avoid encapsulation of *D. suzukii* hemocytes in both in vitro and in vivo assays. Besides, nematode secretions do not seem to play a central role in the lack of encapsulation as we confirmed using dead IJs with unaltered cuticle. Encapsulation was only observed when the cuticle of nematodes was damaged after lipase treatment, suggesting an involvement of the body surface to avoid cellular recognition. Mastore et al. [26] reported similar results demonstrating a lack of encapsulation of alive and dead *S. carpocapsae* IJs in *R. ferrugineus*, although damaged cuticles of nematodes were strongly encapsulated. Furthermore, *S. carpocapsae* avoided the recognition by *G. mellonella* hemocytes while *H. bacteriophora* was recognized [56]. It has been described that *S. carpocapsae* have specific proteins in the epicuticle of IJs that provide a mimetic function to the nematode [23]; besides, in *S. feltiae*, disguise properties could be ascribed to lipids of the epicuticular layer, as suggested by Dunphy and Webster [57]. According to Brivio et al. [22,58], differences observed between *S. carpocapsae* and *S. feltiae* in the immunological relationships with their hosts supported the assumption that EPNs have developed peculiar immunoevasive strategies among different species.

During the in vitro encapsulation assay, a lack of lamellocyte differentiation was observed and resulted in the achievement only of the first steps of an encapsulation process with the attachment of plasmatocytes to lipase-treated nematodes and agarose beads. In contrast, in vivo assays evidenced the lamellocytes differentiation process after nematode infection, causing a decrease of plasmatocytes and an increase of lamellocytes from the constitutive level. The divergence of the differentiation process between assays could be expected due to a lack of natural physiological factors during in vitro assays. Moreover, the cell counts obtained during the in vivo assay showed a high amount of hemocyte populations, in agreement with Kacsoh and Schlenke [59] who suggested that *D. suzukii* larvae had five times more immunocompetent cells than *D. melanogaster*. The results of this assay also confirmed the lack of encapsulation of *S. carpocapsae* despite the differentiation of lamellocytes in the early phase of infection. During the midterm phase of infection, lamellocytes–plasmatocytes aggregation prevented counting hemocytes due to large cellular aggregates whose composition was not identifiable. An important role in the strategy of *S. carpocapsae* is attributable to its secretions of proteases and cytotoxic compounds which induce immunosuppression to the host. Some of these secretions have been identified as serine, cysteine, metallo, and aspartic proteases involved in processes of cell aggregation, clotting response, and cellular apoptosis [25,60]. These authors reported that when *S. carpocapsae* infects *D. melanogaster*, avoids clot enlargement by means of its inhibitor sc-spn6. Unlike plasmatocytes and lamellocytes, our assay crystal cell population remained unaffected even after the nematode released the bacteria. The regular count corroborated the low levels of melanization response observed whereas those cells produced and stored the components of proPO cascade. These results confirmed the different responses of *D. suzukii* larvae to parasitoids as their eggs activate the proPO reaction and cause a strong loss of crystal cells [33]. Moreover, the cellular response of the fly larvae to *L. heterotoma* or *L. boulardi* eggs presented the same pattern with a significant increase of plasmatocytes and lamellocytes [33,59]. In contrast, *D. suzukii* larvae showed a cellular response to *Asobara japonica* Belokobylskij (Hymenoptera: Braconidae) eggs more similar to nematode’s reaction observed in this work, with a slight decrease of plasmatocytes and similar constitutive levels of lamellocytes after long-term parasitization [33].

Our immunological approach aimed to investigate the relationship between an insect pest, such as *D. suzukii* and EPNs to provide an essential understanding of the strategies by which nematobacterial complexes overwhelm the host defenses. The results reported the inhibitory properties of both *S. carpocapsae* and *X. nematophila* to larvae’s immune defenses. Symbiont bacteria affected the humoral response of proPO resulting in lower levels of phenoloxidase activity. In addition, *X. nematophila* infection activated the synthesis of putative AMPs molecules, although their antimicrobial activity was lower than peptides produced from infections with non-entomopathogenic bacteria. Besides, the cell populations of *D. suzukii* were unable to phagocyte the symbiont bacteria or encapsulate *S. carpocapsae* IJs. The data obtained from the encapsulation assays confirmed the elusive properties of the body surface of *S. carpocapsae*. These results attribute to the cuticle a synergistic role with its secretions to prepare an immunologically favorable environment before the release of the symbiont in the hemocoel cavity.

## 5. Conclusions

Along this work, *S. carpocapsae* and *X. nematophila* showed the ability to overtake the immune defenses of *D. suzukii*, therefore confirming the potentiality of this nematode as a biological control agent for this pest. This is the first report that addresses the physiological relationship between EPNs and *D. suzukii* from an immunological aspect; thus, providing a useful starting point to understand the parasite-host relationship between these organisms and help to improve the biological control of *D. suzukii*.

## Figures and Tables

**Table 1:** * Evaluation of hemocytes population (mean ± SD) of total and differential count of three *D. suzukii* larvae treatments: naïve larvae (no infected), early phase of infection (with only nematode presence) and midterm phase of infection (nematode and bacterial presence). Different letters indicate statistically significant differences among treatments for each cell type (*p* < 0.05).

Hemocyte Number/µL of Hemolymph *				
Treatment	Plasmatocytes	Lamellocytes	Crystal Cells	Total Hemocytes
Naїve	25446.88 ± 4358.13 a	195.63 ± 49.88 a	677.50 ± 296.88 a	26320.00 ± 4311.47 a
Early phase	18676.88 ± 3676.56 b	564.38 ± 156.24 b	745.00 ± 184.04 a	19986.25 ± 3733.23 b
Midterm phase	20293.75 ± 3790.60 b	183.75 ± 54.73 a	841.88 ± 341.27 a	21319.38 ± 3791.21 b

## Supplementary Materials

The following are available online at https://www.mdpi.com/2075-4450/11/4/210/s1. Figure S1. Time course of phenoloxidase relative activity in the hemolymph of *D. suzukii*, shown as mean ± SD increase of absorbance recorded every 5 min for 45 min total. Hemolymph samples were from naïve (N), control pricked (CP), *X. nematophila*-infected (InfX), and *E. coli/B. subtilis*-infected (InfB) larvae. Figure S2. The tricine-PAGE pattern of fractioned hemolymph (<30 KDa) extracted from larvae after *X. nematophila* infection. Even though the pattern showed some bands comparable to that observed in *E. coli/M. luteus*-infected larvae, this sample, tested for antimicrobial capability, revealed lower activity compared with that of larvae infected with non-entomopathogenic bacteria. Figure S3. Plated *D. suzukii* hemocytes from naive larvae (**A**) and *X. nematophila* infected-larvae (**B**). Micrographs below (B1, B2, and B3) show the altered morphology of the hemocytes with blebs (arrowheads) protruding from the cell surface. In the micrographs of healthy cells (A) lamellipodia (arrowheads) are visible. Table S1. Statistic comparison of phenoloxidase relative activity in the hemolymph of *D. suzukii* of Figure 1. *P*-value obtained in the Tukey comparison of the hemolymph samples from naïve (N), control pricked (CP), *X. nematophila*-infected (InfX) and *E. coli/B. subtilis*-infected (InfB) larvae, at 0 min and 45 min. Any comparison was considered significant if the *p*-value was < 0.05. Table S2. Statistic comparison of antimicrobial activity in *D. suzukii* hemolymph of Figure 3. *p*-value obtained in the Tukey comparison of the hemolymph samples from naïve (N), control (PBS), *X. nematophila*-infected (InfX), and *E. coli/B. subtilis*-infected (InfB) larvae, in coincubation with *E. coli, M. luteus*, or *X. nematophila*. Any comparison was considered significant if the *p*-value was < 0.05. Table S3. Mortality rate (%) of *E. coli*, *M. luteus*, and *X. nematophila*, when treated with *D. suzukii* hemolymph from larvae infected with InfB or InfX.

## References

[1] Kaya H.K., Gaugler R. (1993). Entomopathogenic nematodes. Annu. Rev. Entomol..

[2] Boemare N., Gaugler R. (2002). Biology, taxonomy, and systematics of *Photorhabdus* and *Xenorhabdus*. Entomopathogenic Nematology.

[3] Snyder H., Stock S.P., Kim S.K., Flores-Lara Y., Forst S. (2007). New insights into the colonization and release processes of *Xenorhabdus nematophila* and the morphology and ultrastructure of the bacterial receptacle of its nematode host, *Steinernema carpocapsae*. Appl. Environ. Microbiol..

[4] Dowds B.C.A., Peters A., Gaugler R. (2002). Virulence mechanisms. Entomopathogenic Nematology.

[5] Lacey L.A., Grzywacz D., Shapiro-Ilan D.I., Frutos R., Brownbridge M., Goettel M.S. (2015). Insect pathogens as biological control agents: Back to the future. J. Invertebr. Pathol..

[6] Brivio M.F., Mastore M. (2018). Nematobacterial complexes and insect hosts: Different weapons for the same war. Insects.

[7] Gaugler R., Wang Y., Campbell J.F. (1994). Aggressive and evasive behaviors in *Popillia japonica* (Coleoptera, Scarabaeidae) larvae—defenses against entomopathogenic nematode attack. J. Invertebr. Pathol..

[8] Castillo J.C., Reynolds S.E., Eleftherianos I. (2011). Insect immune responses to nematode parasites. Trends Parasitol..

[9] Eleftherianos I., Shokal U., Yadav S., Kenney E., Maldonado T. (2017). Insect immunity to entomopathogenic nematodes and their mutualistic bacteria. Curr. Top. Microbiol. Immunol..

[10] Gillespie J.P., Kanost M.R., Trenczek T. (1997). Biological mediators of insect immunity. Annu. Rev. Entomol..

[11] Kim C.H., Park J.W., Ha N.C., Kang H.J., Lee B.L. (2008). Innate immune response in insects: Recognition of bacterial peptidoglycan and amplification of its recognition signal. BMB Rep..

[12] Nappi A.J., Kohler L., Mastore M. (2004). Signaling pathways implicated in the cellular innate immune responses of *Drosophila*. Invertebr. Surv. J..

[13] Scherfer C., Karlsson C., Loseva O., Bidla G., Goto A., Havemann J., Dushay M.S., Theopold U. (2004). Isolation and Characterization of Hemolymph Clotting Factors in *Drosophila melanogaster* by a Pullout Method. Curr. Biol..

[14] Dziedzeich A., Shivankar S., Theopold U. (2020). *Drosophila melanogaster* Responses against Entomopathogenic Nematodes: Focus on Hemolymph Clots. Insects.

[15] Bulet P., Stöcklin R. (2005). Insect antimicrobial peptides: Structures, properties and gene regulation. Protein Pept. Lett..

[16] Strand M.R. (2008). The insect cellular immune response. Insect Sci..

[17] De Lerma Barbaro A., Gariboldi M.B., Mastore M., Brivio M.F., Giovannardi S. (2019). In Vivo Effects of A Pro-PO System Inhibitor on the Phagocytosis of *Xenorhabdus nematophila* in *Galleria mellonella* Larvae. Insects.

[18] Lemaitre B., Hoffmann J. (2007). The host defense of *Drosophila melanogaster*. Annu. Rev. Immunol..

[19] Letourneau M., Lapraz F., Sharma A., Vanzo N., Waltzer L., Crozatier M. (2016). *Drosophila* hematopoiesis under normal conditions and in response to immune stress. FEBS Lett..

[20] Rizki T., Rizki R., Grell E. (1980). A mutant affecting the crystal cells in *Drosophila melanogaster*. Roux’s Arch. Dev. Biol..

[21] Binda-Rossetti S., Mastore M., Protasoni M., Brivio M.F. (2016). Effects of an entomopathogen nematode on the immune response of the insect pest red palm weevil: Focus on the host antimicrobial response. J. Invertebr. Pathol..

[22] Brivio M.F., Mastore M., Nappi A.J. (2010). A pathogenic parasite interferes with phagocytosis of insect immunocompetent cells. Dev. Comp. Immunol..

[23] Wang Y., Gaugler R. (1999). *Steinernema glaseri* surface coat protein suppresses the immune response of *Popillia japonica* (Coleoptera: Scarabaeidae) larvae. Biol. Control.

[24] Mastore M., Brivio M.F. (2008). Cuticular surface lipids are responsible for disguise properties of an entomoparasite against host cellular responses. Dev. Comp. Immunol..

[25] Toubarro D., Avila M.M., Hao Y., Balasubramanian N., Jing Y., Montiel R., Faria T.Q., Brito R.M., Simões N. (2013). A serpin released by an entomopathogen impairs clot formation in insect defense system. PLoS ONE.

[26] Mastore M., Arizza V., Manachini B., Brivio M.F. (2015). Modulation of immune responses of *Rhynchophorus ferrugineus* (Insecta: Coleoptera) induced by the entomopathogenic nematode *Steinernema carpocapsae* (Nematoda: Rhabditida). Insect Sci..

[27] Brivio M.F., Toscano A., Pasquale S.M., Barbaro A.D., Giovannardi S., Finzi G., Mastore M. (2018). Surface protein components from entomopathogenic nematodes and their symbiotic bacteria: Effects on immune responses of the greater wax moth, *Galleria mellonella* (Lepidoptera: Pyralidae). Pest Manag. Sci..

[28] Park Y., Kim Y. (2003). *Xenorhabdus nematophilus* inhibits p-bromophenacyl bromide (BPB)-sensitive PLA2 of *Spodoptera exigua*. Arch. Insect Biochem. Physiol..

[29] Walsh D.B., Bolda M.P., Goodhue R.E., Dreves A.J., Lee J., Bruck D.J., Walton V.M., O’Neal S.D., Zalom F.G. (2011). *Drosophila suzukii* (Diptera: Drosophilidae): Invasive Pest of Ripening Soft Fruit Expanding its Geographic Range and Damage Potential. J. Integr. Pest Manag..

[30] Haye T., Girod P., Cuthbertson A.G.S., Wang X.G., Daane K.M., Hoelmer K.A., Baroffio C., Zhang J.P., Desneux N. (2016). Current SWD IPM tactics and their practical implementation in fruit crops across different regions around the world. J. Pest Sci..

[31] Lee J.C., Wang X., Daane K.M., Hoelmer K.A., Isaacs R., Sial A.A., Walton V.M. (2019). Biological control of spotted-wing drosophila—current and pending tactics. J. Integr. Pest Manag..

[32] Chabert S., Allemand R., Poyet M., Eslin P., Gibert P. (2012). Ability of European parasitoids (Hymenoptera) to control a new invasive Asiatic pest, *Drosophila suzukii*. Biol. Control.

[33] Poyet M., Havard S., Prevost G., Chabrerie O., Doury G., Gibert P., Eslin P. (2013). Resistance of *Drosophila suzukii* to the larval parasitoids *Leptopilina heterotoma* and *Asobara japonica* is related to haemocyte load. Physiol. Entomol..

[34] Stacconi M.V., Amiresmaeili N., Biondi A., Carli C., Caruso S., Dindo M.L., Francati S., Gottardello A., Grassi A., Lupi D. (2018). Host location and dispersal ability of the cosmopolitan parasitoid *Trichopria drosophilae* released to control the invasive spotted wing *Drosophila*. Biol. Control.

[35] Yousef M., Aranda-Valera E., Quesada-Moraga E. (2018). Lure-and-infect and lure-and-kill devices based on *Metarhizium brunneum* for spotted wing *Drosophila* control. J. Pest Sci..

[36] Garriga A., Morton A., Garcia-del-Pino F. (2018). Is *Drosophila suzukii* as susceptible to entomopathogenic nematodes as *Drosophila melanogaster*?. J. Pest Sci..

[37] Garriga A., Morton A., Ribes A., Garcia-del-Pino F. (2020). Soil emergence of *Drosophila suzukii* adults: A susceptible period for entomopathogenic nematodes infection. J. Pest Sci..

[38] Woodring J.L., Kaya H.K. (1998). Steinernematid and heterorhabditid nematodes: A handbook of techniques. South. Coop. Bull..

[39] White G.F. (1927). A method for obtaining infective nematode larvae from cultures. Science.

[40] Park Y., Kim Y. (2000). Eicosanoids rescue *Spodoptera exigua* infected with *Xenorhabdus nematophilus*, the symbiotic bacteria to the entomopathogenic nematode *Steinernema carpocapsae*. J. Insect Physiol..

[41] Mastore M., Brivio M.F., Sandrelli F., Tettamanti G. (2020). Basic Methods to Evaluate Humoral Immunity Processes in Lepidoptera Larvae. Immunity in Insects. Springer Protocols Handbooks.

[42] Schägger H., Von Jagow H. (1987). Tricine-sodium dodecyl sulfate-polyacrylamide gel electrophoresis for the separation of proteins in the range from 1 to 100 kDa. Anal. Biochem..

[43] Arefin B., Kucerova L., Krautz R., Kranenburg H., Parvin F., Theopold U. (2015). Apoptosis in hemocytes induces a shift in effector mechanisms in the *Drosophila* immune system and leads to a pro-inflammatory state. PLoS ONE.

[44] R Core Team (2017). R: A Language and Environment for Statistical Computing.

[45] Peña J.M., Carrillo M.A., Hallem E.A. (2015). Variation in the susceptibility of *Drosophila* to different entomopathogenic nematodes. Infect. Immun..

[46] Kim Y.G., Shrestha S., Song C.J., Seo S.Y. (2011). Bacterial metabolites of an entomopathogenic bacterium, *Xenorhabdus nematophila*, inhibit a catalytic activity of phenoloxidase of the diamondback moth, *Plutella xylostella*. J. Microbiol. Biotechnol..

[47] Scarpati M., Qi Y., Govind S., Singh S. (2019). A combined computational strategy of sequence and structural analysis predicts the existence of a functional eicosanoid pathway in *Drosophila melanogaster*. PLoS ONE.

[48] Mastore M., Quadroni S., Toscano A., Mottadelli N., Brivio M.F. (2019). Susceptibility to entomopathogens and modulation of basal immunity in two insect models at different temperatures. J. Therm. Biol..

[49] Daffre S., Kylsten P., Samakovlis C., Hultmark D. (1994). The lysozyme locus in *Drosophila melanogaster*: An expanded gene family adapted for expression in the digestive tract. Mol. Gen. Genet..

[50] Ji D., Kim Y. (2004). An entomopathogenic bacterium, *Xenorhabdus nematophila*, inhibits the expression of an antibacterial peptide, cecropin, of the beet armyworm, *Spodoptera exigua*. J. Insect Physiol..

[51] Duvic B., Jouan V., Essa N., Girard P.A., Pages S., Khattar Z.A., Volkoff N.A., Givaudan A., Destoumieux-Garzón D., Escoubas J.M. (2012). Cecropins as a marker of *Spodoptera frugiperda* immunosuppression during entomopathogenic bacterial challenge. J. Insect Physiol..

[52] Mastore M., Binda Rossetti S., Giovannardi S., Scarì G., Brivio M.F. (2015). Inducible factors with antimicrobial activity after immune challenge in the haemolymph of Red Palm Weevil (Insecta). Innate Immun..

[53] McQuade R., Stock S.P. (2018). Secretion systems and secreted proteins in gram-negative entomopathogenic bacteria: Their roles in insect virulence and beyond. Insects.

[54] Shrestha S., Kim Y. (2007). An entomopathogenic bacterium, *Xenorhabdus nematophila*, inhibits hemocyte phagocytosis of *Spodoptera exigua* by inhibiting phospholipase A_2_. J. Invertebr. Pathol..

[55] Au C., Dean P., Reynolds S.E., Ffrench-Constant R.H. (2004). Effect of the insect pathogenic bacterium *Photorhabdus* on insect phagocytes. Cell. Microbiol..

[56] Ebrahimi L., Niknam G., Dunphy G.B. (2011). Hemocyte responses of the Colorado potato beetle, *Leptinotarsa decemlineata*, and the greater wax moth, *Galleria mellonella*, to the entomopathogenic nematodes, *Steinernema feltiae* and *Heterorhabditis bacteriophora*. J. Insect Sci..

[57] Dunphy G., Webster J. (1987). Partially characterized components of the epicuticle of dauer juvenile *Steinernema feltiae* and their influence on the hemocyte activity in *Galleria mellonella*. J. Parasitol..

[58] Brivio M.F., Mastore M., Moro M. (2004). The role of *Steinernema feltiae* body-surface lipids in host–parasite immunological interactions. Mol. Biochem. Parasitol..

[59] Kacsoh B.Z., Schlenke T.A. (2012). High hemocyte load is associated with increased resistance against parasitoids in *Drosophila suzukii*, a relative of *D. melanogaster*. PLoS ONE.

[60] Balasubramanian N., Hao Y.J., Toubarro D., Nascimento G., Simões N. (2009). Purification, biochemical and molecular analysis of a chymotrypsin protease with prophenoloxidase suppression activity from the entomopathogenic nematode *Steinernema carpocapsae*. Int. J. Parasitol..

