# Supplementary material for: Immune Response of Drosophila suzukii Larvae to Infection with the Nematobacterial Complex Steinernema carpocapsae–Xenorhabdus nematophila
Source: Insects. 2020 Mar 28;11(4):210. doi: 10.3390/insects11040210 (PMC7240654; doi:10.3390/insects11040210)
Supplement: Supplementary file 1 [file insects-11-00210-s001.pdf]

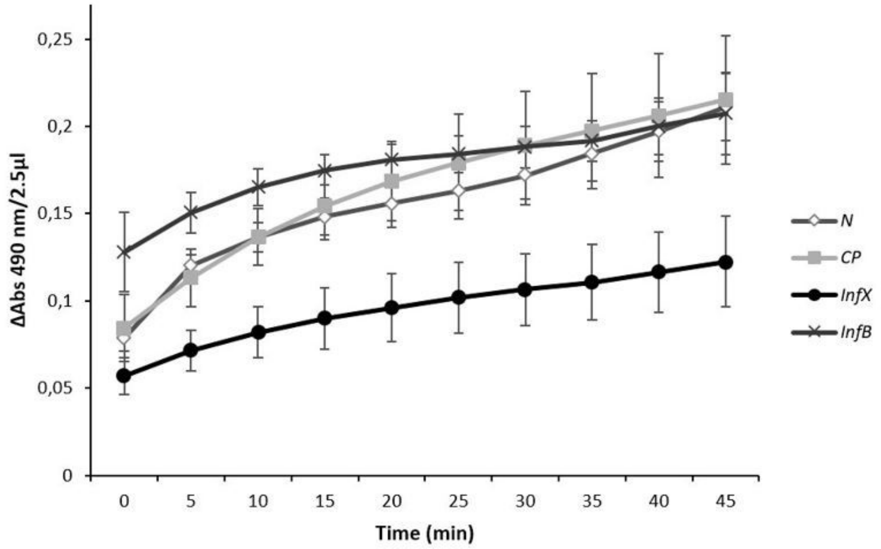

Figure S1. Time course of phenoloxidase relative activity in the hemolymph of *D. suzukii*, shown as mean ± SD increase of absorbance recorded every 5 min for 45 min total. Hemolymph samples were from naïve (N), control pricked (CP), *X. nematophila*-infected (InfX), and *E. coli*/*B. subtilis*-infected (InfB) larvae.

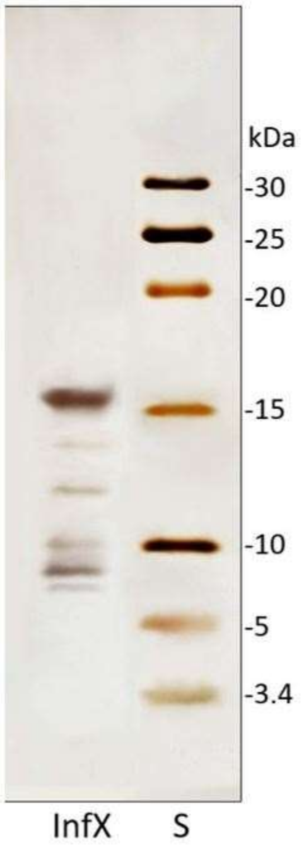

Figure S2. The tricine-PAGE pattern of fractioned hemolymph (<30 KDa) extracted from larvae after *X. nematophila* infection. Even though the pattern showed some bands comparable to that observed in *E. coli*/*M. luteus*-infected larvae, this sample, tested for antimicrobial capability, revealed lower activity compared with that of larvae infected with non-entomopathogenic bacteria.

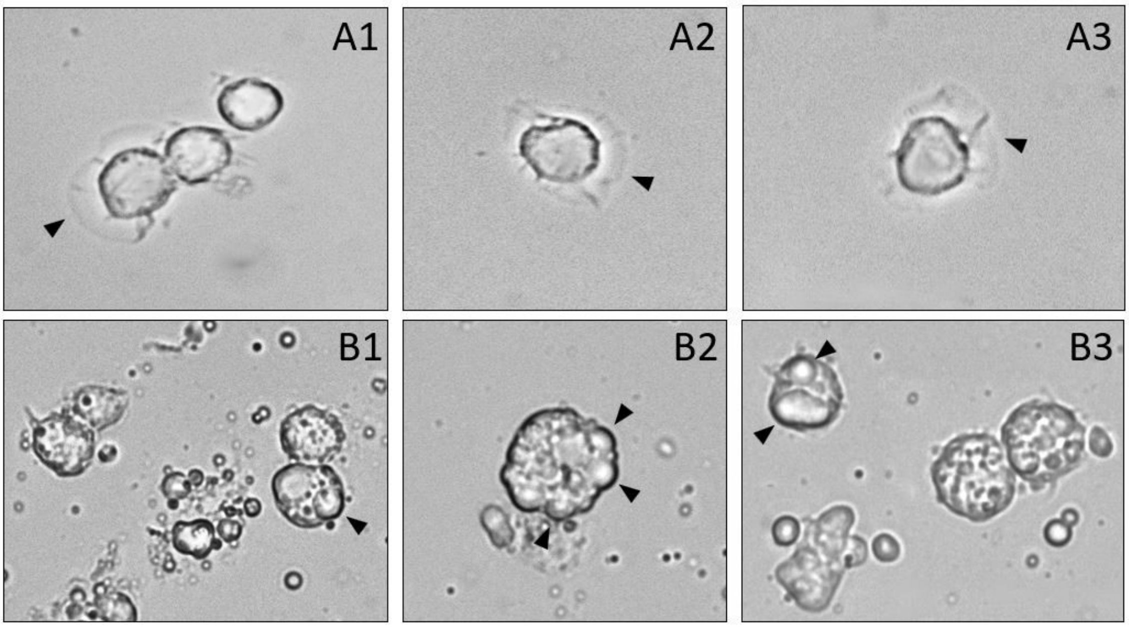

Figure S3. Plated *D. suzukii* hemocytes from naive larvae (**A**) and *X. nematophila* infected-larvae (**B**). Micrographs below (B1, B2, and B3) show the altered morphology of the hemocytes with blebs (arrowheads) protruding from the cell surface. In the micrographs of healthy cells (A) lamellipodia (arrowheads) are visible.

Table S1. Statistic comparison of phenoloxidase relative activity in the hemolymph of *D. suzukii* of Figure 1. *P*-value obtained in the Tukey comparison of the hemolymph samples from naïve (N), control pricked (CP), *X. nematophila*-infected (InfX) and *E. coli*/*B. subtilis*-infected (InfB) larvae, at 0 min and 45 min. Any comparison was considered significant if the *p*-value was < 0.05.

Tukey Comparison	*p*-Value at 0 min	*p*-Value at 45 min
CP—InfB	0.0030	0.9630
CP—N	0.9320	0.9940
CP—InfX	0.0670	0.0003
InfB—N	0.0010	0.9950
InfB—InfX	0.0001	0.0008
N—InfX	0.1930	0.0005

Table S2. Statistic comparison of antimicrobial activity in *D. suzukii* hemolymph of Figure 3. *p*-value obtained in the Tukey comparison of the hemolymph samples from naïve (N), control (PBS), *X. nematophila*-infected (InfX), and *E. coli*/*B. subtilis*-infected (InfB) larvae, in coincubation with *E. coli, M. luteus*, or *X. nematophila*. Any comparison was considered significant if the *p*-value was < 0.05.

Tukey Comparison	*p*-Value for *E. coli*	*p*-Value for *M. luteus*	*p*-Value for *X. nematophila*
PBS—InfB	0.077	0.9910	0.188
PBS—N	0.250	0.0006	0.035
PBS— InfX	0.804	0.0720	-
InfB—N	0.005	0.0005	0.269
InfB—InfX	0.265	0.0480	-
N—InfX	0.072	0.0200	-

Table S3. Mortality rate (%) of *E. coli, M. luteus,* and *X. nematophila,* when treated with *D. suzukii* hemolymph from larvae infected with InfB or InfX. T.

Bacteria Mortality (%)		
**Bacteria**	**InfX**	**InfB**
E. coli	55.0%	92.2%
M. luteus	41.5%	76.1%
X. nematophila	n.d.	17.2%
